# Supplementary material for: Content-rich biological network constructed by mining PubMed abstracts
Source: BMC Bioinformatics. 2004 Oct 8;5:147. doi: 10.1186/1471-2105-5-147 (PMC528731; doi:10.1186/1471-2105-5-147)
Supplement: Additional File 2 — The original results of the above study (non-essential files are deleted to keep the file size under the limit set by BMC bioinformatics). [file 1471-2105-5-147-S2.bz2 › chilibotAdditionalFile2/dip40/459ID10488147E10477/html/CX3CL1_CX3CR1.html]

 


 **CX3CL1** and **CX3CR1** 
  
Found 71 abstracts in PubMed, retrieved 40.  
 

 What does Google say? 
 PDF only 
| .edu only 

---

**Interactive relationship** (e.g. stimulation, inhibition, etc)

**Neutral relationship**- CONCLUSION Our findings suggest that the interactions of  **CX3CL1**  and  **CX3CR1**  might contribute to the accumulation of  **CX3CR1**  T cells expressing type 1 cytokines and possessing cytotoxic granules in RA synovium.  Ref: 12428227 Arthritis Rheum, 2002
- The chemokine receptor  **CX3CR1**  is a proinflammatory leukocyte receptor specific for the chemokine fractalkine  [ **CX3CL1** ]  FKN or  **CX3CL1** .  Ref: 12697743 J Clin Invest, 2003
- This membrane bound localization allows fractalkine  [ **CX3CL1** ]  to function as an adhesion molecule for cells bearing its receptor,  **CX3CR1** .  Ref: 11495925 J Biol Chem, 2001
- Dual functions of fractalkine  [ **CX3CL1** ]  CX3C  [ **CX3CL1** ]  ligand 1 in trafficking of perforin granzyme B cytotoxic effector lymphocytes that are defined by  **CX3CR1**  expression.  Ref: 12055230 J Immunol, 2002
- BACKGROUND Fractalkine  [ **CX3CL1** ]  is a novel chemokine that mediates both firm adhesion of leukocytes to the endothelium via  **CX3CR1**  and leukocyte transmigration out of the bloodstream.  Ref: 12459434 Cardiovasc Pathol< MedlineTA>Cardiovasc Pathol, 2002
- Fractalkine  [ **CX3CL1** ]  is a neuronally expressed chemokine that acts through its G protein coupled receptor  **CX3CR1** , localized on microglial and immune cells.  Ref: 12059974 Eur J Neurosci, 2002
- The human hepatocarcinoma cell line HepG2 expressed fractalkine  [ **CX3CL1** ]  at the gene and protein level, and HepG2 conditioned medium was chemotactic for cells overexpressing  **CX3CR1** .  Ref: 12076860 J Hepatol, 2002
- Fractalkine  [ **CX3CL1** ] , a chemokine expressed by inflamed endothelium, induces leukocyte adhesion and migration via the receptor  **CX3CR1** , and the  **CX3CR1**  polymorphism V249I affects receptor expression and function.  Ref: 11532900 Circ Res, 2001
- **CX3CR1**  staining was seen in the coronary arteries of all cases, with specific localization to regions with fractalkine  [ **CX3CL1** ]  staining.  Ref: 12459434 Cardiovasc Pathol< MedlineTA>Cardiovasc Pathol, 2001
- In contrast to CD16 monocytes, CD16 monocytes expressed high  **CX3CR1**  and CXCR4 but low CCR2 and CD62L levels and underwent efficient transendo thelial migration in response to fractalkine  [ **CX3CL1** ]  FKN.  Ref: 12810688 J Exp Med, 2003
- BACKGROUND Fractalkine  [ **CX3CL1** ]   **CX3CL1** , a CX3C  [ **CX3CL1** ]  chemokine, is expressed in the vessel wall and mediates the firm adhesion and chemotaxis of leukocytes expressing its receptor,  **CX3CR1** .  Ref: 12600915 Circulation, 2003
- Decreased atherosclerosis in  **CX3CR1**  mice reveals a role for fractalkine  [ **CX3CL1** ]  in atherogenesis.  Ref: 12569158 J Clin Invest, 2003
- Fractalkine  [ **CX3CL1** ] , SDF 1alpha, RANTES and MDC inhibited the I Ba in  **CX3CR1** , CXCR4, CCR5 and CCR4 expressing G1A1 cells, respectively.  Ref: 11880151 J Neuroimmunol, 2002
- While soluble fractalkine  [ **CX3CL1** ]  present in SMC conditioned medium is capable of inducing calcium transients in cells expressing the fractalkine  [ **CX3CL1** ]  receptor  **CX3CR1** , blocking experiments using neutralizing Abs reveal that it can be inactivated without affecting the chemotactic activity of SMC conditioned media on monocytes.  Ref: 11777952 J Immunol, 2002
- In a prion model of chronic neurodegeneration and inflammation,  **CX3CL1**  immunoreactivity is upregulated in astrocytes and  **CX3CR1**  expression is elevated on microglia.  Ref: 11870871 Glia, 2002

**Non-interactive relationship** (e.g. studied together, co-existance, homology, etc.)

- The fraction of CD45RO and CD45RO T helper Th cells expressing the fractalkine  [ **CX3CL1** ]  receptor  **CX3CR1**  was higher in HIV infected patients than in healthy individuals, and these cells were abnormally sensitive to fractalkine  [ **CX3CL1** ]  stimulation.  Ref: 11535497 Blood, 2001
- We evaluated by in situ hybridization and immunohistochemistry the expression of fractalkine  [ **CX3CL1** ]  and  **CX3CR1**  in the rat brain, after a transient occlusion of the middle cerebral artery.  Ref: 12059974 Eur J Neurosci, 2002
- Transcripts for  **CX3CR1**  were detected in HepG2, and exposure of these cells to recombinant fractalkine  [ **CX3CL1** ]  induced cell migration.  Ref: 12076860 J Hepatol, 2002
- Consistent with this, intravenous injection of a single dose of recombinant IL 15 into C57BL 6 mice decreased steady state  **CX3CR1**  levels 24 hours after injection in freshly isolated PBMCs, splenocytes and bone marrow cells, and treatment of mouse PBMCs with IL 15 in vitro inhibited  **CX3CL1**  induced chemotaxis.  Ref: 12881312 BloodBlood, 2002
- To explore the relationship between TGF beta1 and  **CX3CR1**  in the CNS, the effects of TGF beta1 on  **CX3CR1**  mRNA, protein and fractalkine  [ **CX3CL1** ]  dependent stimulation of signal transduction cascades in primary cultures of rat microglia were examined.  Ref: 12446007 J Neuroimmunol, 2002
- METHOD Polyclonal rabbit antibodies were used to immunostain human fractalkine  [ **CX3CL1** ]  and  **CX3CR1**  to localize their presence in transverse sections of the proximal left anterior descending and or right coronary arteries.  Ref: 12459434 Cardiovasc Pathol< MedlineTA>Cardiovasc Pathol, 2002
- Immunohistochemistry was performed using primary antibodies recognizing soluble and membrane bound  **CX3CL1**  and the N terminus of the  **CX3CR1** .  Ref: 11870871 Glia, 2002
- TGF beta1 increased steady state levels of  **CX3CR1**  mRNA, 125I fractalkine  [ **CX3CL1** ]  binding sites and blunted fractalkine  [ **CX3CL1** ]  stimulated ERK1 2 phosphorylation.  Ref: 12446007 J Neuroimmunol, 2002
- In this study, we characterized the presence of fractalkine  [ **CX3CL1** ]  and its receptor  **CX3CR1**  in human coronary arteries from normal, atherosclerotic, diabetic, and TVD settings.  Ref: 12459434 Cardiovasc Pathol< MedlineTA>Cardiovasc Pathol, 2002
- Fractalkine  [ **CX3CL1** ]  expression and the recruitment of  **CX3CR1**  cells in the prolonged mesangial proliferative glomerulonephritis.  Ref: 12028445 Kidney Int, 2002
- CONCLUSIONS Fractalkine  [ **CX3CL1** ]  expression and the recruitment of  **CX3CR1**  cells in glomeruli might play an important role in the development of the prolonged disease.  Ref: 12028445 Kidney Int, 2002
- In this study, we examined the role of  **CX3CL1**  fractalkine  [ **CX3CL1** ]  and its receptor CX3C  [ **CX3CL1** ]  chemokine receptor 1  [ **CX3CR1** ]   **CX3CR1**  in the T cell migration into RA synovium.  Ref: 12428227 Arthritis Rheum, 2002
- The expression of  **CX3CL1**  and  **CX3CR1**  by glial cells during inflammation in the CNS may be influenced by the surrounding cytokine milieu, which has been shown to differ in acute and chronic neuroinflammation.  Ref: 11870871 Glia, 2002
- Both fractalkine  [ **CX3CL1** ]  and  **CX3CR1**  were expressed constitutively in neurons, microglia, and astrocytes.  Ref: 12850572 Brain Res, 2003
- CONCLUSIONS The  **CX3CR1**   **CX3CL1**  pathway seems to play a direct and critical role in monocyte recruitment and atherosclerotic lesion development in a mouse model of human atherosclerosis.  Ref: 12600915 Circulation, 2003
- We found that neurons in the naive rodent brain are immunoreactive for  **CX3CL1**  and  **CX3CR1** , both showing a perinuclear staining pattern.  Ref: 11870871 Glia, 2002
- CONCLUSION The distinctive staining patterns in native atherosclerosis, diabetes mellitus with atherosclerosis, and TVD indicate that the expression of fractalkine  [ **CX3CL1** ]  and  **CX3CR1**  may be important in the pathogenesis of these diseases.  Ref: 12459434 Cardiovasc Pathol< MedlineTA>Cardiovasc Pathol, 2002
- OBJECTIVE To investigate the expression of fractalkine  [ **CX3CL1** ]  FKN and its receptor  **CX3CR1**  in cardiac allografts and the effect of Cyclosporin A CsA.  Ref: 12783679 Zhonghua Wai Ke Za Zhi, 2003
- Expression of fractalkine  [ **CX3CL1** ]   **CX3CL1**  and its receptor,  **CX3CR1** , during acute and chronic inflammation in the rodent CNS.  Ref: 11870871 Glia, 2002
- The aim of this study was to investigate the expression patterns of fractalkine  [ **CX3CL1** ]  and its receptor  **CX3CR1**  in normal human liver and in conditions of injury.  Ref: 12076860 J Hepatol, 2002
- TGF beta1 upregulates  **CX3CR1**  expression and inhibits fractalkine  [ **CX3CL1** ]  stimulated signaling in rat microglia.  Ref: 12446007 J Neuroimmunol, 2002
- In this study, we investigate the expression of fractalkine  [ **CX3CL1** ]   **CX3CL1**  and the fractalkine  [ **CX3CL1** ]  receptor  **CX3CR1**  in the naive rat and mouse central nervous system CNS.  Ref: 11870871 Glia, 2002
- Up regulated expression of fractalkine  [ **CX3CL1** ]  and its receptor  **CX3CR1**  during liver injury in humans.  Ref: 12076860 J Hepatol, 2002
- METHODS Using flow cytometry, immunohistochemistry, and reverse transcription polymerase chain reaction, we analyzed  **CX3CR1**  expression by peripheral blood and synovial T cells, and  **CX3CL1**  expression in synovium from patients with RA.  Ref: 12428227 Arthritis Rheum, 2002
- AIMS In this study, we determined the signal transduction and functional consequences after ligand specific activation of the fractalkine  [ **CX3CL1** ]  receptor  **CX3CR1**  in human intestinal epithelial cells.  Ref: 11781291 Gastroenterology, 2002
- Fractalkine  [ **CX3CL1** ]  has been identified as a novel chemokine that exhibits cell adhesion and chemoattractive properties in the central nervous system CNS, and the fractalkine  [ **CX3CL1** ]  receptors,  **CX3CR1** , are also expressed in the CNS.  Ref: 12125082 J Neurosci Res, 2002
- Ad35K infected cell culture medium, containing recombinant 35K, potently reduced migration of CCR5 transfected 293 cells by 95% in response to the CC chemokine RANTES, but had no effect on cells transfected with the  **CX3CR1**  fractalkine  [ **CX3CL1** ]  receptor.  Ref: 12777060 Immunobiology, 2003
- RESULTS Both fractalkine  [ **CX3CL1** ]  and  **CX3CR1**  were up regulated during chronic injury, in areas of portal and lobular inflammation.  Ref: 12076860 J Hepatol, 2002
- In severe acute hepatitis, fractalkine  [ **CX3CL1** ]  and  **CX3CR1**  were expressed at high levels not only in areas of inflammation but also in regenerating epithelial cells within bile duct like structures, which showed co expression of fractalkine  [ **CX3CL1** ]  and cytokeratin 7 or  **CX3CR1** .  Ref: 12076860 J Hepatol, 2002
- Fractalkine  [ **CX3CL1** ]   **CX3CL1**  is a chemokine which is expressed predominantly in the central nervous system, being localized on neurons, while its receptor,  **CX3CR1** , is found on microglial cells.  Ref: 12725801 Methods, 2003
- Upon binding to the CX3C  [ **CX3CL1** ]  receptor one  **CX3CR1** , FKN induces adhesion, chemoattraction, and activation of leukocytes, including brain macrophages and microglia MP.  Ref: 12476352 J Neurovirol, 2002
- Expression of fractalkine  [ **CX3CL1** ]  and the number of the fractalkine  [ **CX3CL1** ]  receptor  **CX3CR1**  positive cells in the glomeruli in the prolonged model were significantly higher than those in the reversible model.  Ref: 12028445 Kidney Int, 2002
- Characterization of fractalkine  [ **CX3CL1** ]   **CX3CL1**  and  **CX3CR1**  in human coronary arteries with native atherosclerosis, diabetes mellitus, and transplant vascular disease.  Ref: 12459434 Cardiovasc Pathol< MedlineTA>Cardiovasc Pathol, 2002
- Then, the localization of fractalkine  [ **CX3CL1** ]  and the characteristics of  **CX3CR1**  cells were analyzed in glomeruli.  Ref: 12028445 Kidney Int, 2002
- **CX3CR1**  tyrosine sulfation enhances fractalkine  [ **CX3CL1** ]  induced cell adhesion.  Ref: 11909868 J Biol Chem, 2002
- Although the treatment did not ameliorate the morphological finding at two weeks, decreased expression of fractalkine  [ **CX3CL1** ]  and  **CX3CR1**  were already detected at two weeks in rats treated with candesartan.  Ref: 12028445 Kidney Int, 2002
- **CX3CL1** , the unique ligand of  **CX3CR1** , was expressed by endothelial cells and synoviocytes in RA synovium, but not in osteoarthritis synovium.  Ref: 12428227 Arthritis Rheum, 2002
- BACKGROUND  **CX3CL1**  fractalkine  [ **CX3CL1** ]  is a membrane bound chemokine that can function as an adhesion molecule for cells expressing the receptor  **CX3CR1** .  Ref: 12110009 Kidney Int, 2002
- In the present study, we examined the production of fractalkine  [ **CX3CL1** ]  and the expression of its receptor,  **CX3CR1**  in murine glial and neuronal cell in vitro, and investigated its neuroprotective functions.  Ref: 12850572 Brain Res, 2003
- In two examples, we showed that chemokines influence notably the cellular immune functions, such as CD8 cytotoxicity Rantes CCR3 and gamma interferon production fractalkine  [ **CX3CL1** ]   **CX3CR1** .  Ref: 12134637 J Soc Biol, 2002
- Expression of the fractalkine  [ **CX3CL1** ]  receptor  **CX3CR1**  in human kidney diseases.  Ref: 12110009 Kidney Int, 2002
- Transient transfections with dnIkappaB alpha, dnIkappaB beta, dnIKK gamma, kdNIK, and kdIKK beta or pretreatment with wortmannin, Akt inhibitor, PDTC, or MG 132 significantly attenuated TNF alpha induced fractalkine  [ **CX3CL1** ]  and  **CX3CR1**  expression.  Ref: 12729461 Biochem J< MedlineTA>Biochem J, 2002
- We have previously shown that reduced expression of the fractalkine  [ **CX3CL1** ]  receptor,  **CX3CR1** , is correlated with rapid HIV disease progression and with reduced susceptibility to acute coronary events.  Ref: 12234253 Biochem J, 2002
- Expression of fractalkine  [ **CX3CL1** ]  and its receptor,  **CX3CR1** , in response to ischaemia reperfusion brain injury in the rat.  Ref: 12059974 Eur J Neurosci, 2002
- Interactions between fractalkine  [ **CX3CL1** ]  and  **CX3CR1**  can mediate not only chemotaxis but also cell adhesion in the absence of substrates for other adhesion molecules.  Ref: 12806431 Drug News Perspect, 2001
- Acute inflammatory responses in the CNS, induced by stereotaxic injections of lipopolysaccharide or kainic acid, results in activation of microglia and astrocytes but no detectable changes in the glial expression of  **CX3CL1**  or  **CX3CR1** .  Ref: 11870871 Glia, 2002
- Fractalkine  [ **CX3CL1** ]  receptor  **CX3CR1**  is expressed on natural killer NK cells, monocytes and some portions of CD8 T cells.  Ref: 12806431 Drug News Perspect, 2001
- OBJECTIVESWe have recently reported that the polymorphism of the fractalkine  [ **CX3CL1** ]  receptor,  **CX3CR1** , provides a new marker for prognosis in HIV disease.  Ref: 12799549 AIDS, 2003
- Studies using a static model of adhesion demonstrated  **CX3CR1**  fractalkine  [ **CX3CL1** ]  interactions accounted for 26% of monocytic THP 1 cell and 17% of peripheral blood natural killer cell adhesion to tubular epithelial cells, suggesting that fractalkine  [ **CX3CL1** ]  may have a functional role in leucocyte adhesion and retention, at selected tubular sites in acute renal inflammation.  Ref: 12100035 Clin Exp Immunol, 2002
- Pretreatment with pertussis toxin or anti  **CX3CR1**  neutralizing antibodies attenuated TNF alpha induced fractalkine  [ **CX3CL1** ]  induction indicating that fractalkine  [ **CX3CL1** ]  autoregulation plays a role in TNF alpha induced sustained fractalkine  [ **CX3CL1** ]  expression.  Ref: 12729461 Biochem J< MedlineTA>Biochem J, 2002
- Fractalkine  [ **CX3CL1** ]  CX3C  [ **CX3CL1** ]  ligand 1 and its receptor  **CX3CR1**  are known to mediate both cell adhesion and cell migration.  Ref: 12055230 J Immunol, 2002
- TNF alpha activated NF gammaB and induced fractalkine  [ **CX3CL1** ]  and  **CX3CR1**  expression in a time dependent manner in rat aortic smooth muscle cells.  Ref: 12729461 Biochem J< MedlineTA>Biochem J, 2002
